# A Case of Severe Metabolic Acidosis in the Setting of a Strict Ketogenic Diet

**DOI:** 10.7759/cureus.38741

**Published:** 2023-05-08

**Authors:** Kerrie E Ward, Jay Ramsay, Bao Joseph Vu

**Affiliations:** 1 Internal Medicine, University of California Irvine, Orange, USA; 2 Internal Medicine, Veterans Affairs Long Beach Healthcare System, Long Beach, USA

**Keywords:** refeeding syndrome, electrolyte abnormalities, starvation ketoacidosis, metabolic acidosis, ketogenic diet

## Abstract

Patients with metabolic acidosis often present with obscure, multifactorial etiologies, making efficient diagnosis and treatment key to preventing poor clinical outcomes. This case report describes a patient with severe metabolic acidosis in which the underlying cause was not immediately apparent. After a thorough work-up and history taking, the patient’s strict ketogenic diet was identified as the most likely source of his illness. Over multiple days, the patient improved as he resumed a normal diet and was treated for refeeding syndrome. This case highlights the importance of taking a comprehensive social and diet history when assessing a patient with metabolic acidosis. It also highlights the need for physicians to understand and be ready to counsel on the possible effects of fad diets, such as the ketogenic diet.

## Introduction

Metabolic acidosis is a life-threatening condition that must be assessed in a systematic and timely fashion. While there are a myriad of etiologies, they all result in the lowering of the blood pH and stressing the body’s ability to maintain homeostasis. The pathogenesis of metabolic acidosis includes three major mechanisms: increased acid generation, decreased acid secretion by the kidneys, and loss of bicarbonate. These mechanisms can help to distinguish the underlying cause and direct therapy. Additionally, the calculation of the anion gap and osmolal gap can help narrow the wide differential [1].

However, in clinical practice, the underlying diagnosis is often much less black and white and requires a thorough medical work-up to assess for underlying disease such as diabetes or renal disease, along with thorough history taking if the patient is alert and oriented. This case report describes a patient whose clinical picture and workup did not initially fit any of the most common causes of metabolic acidosis, and was found to have an unexpected, self-induced etiology.

## Case presentation

A 58-year-old male with a history of hyperlipidemia and resolved hepatitis B infection presented to the emergency department with progressive fatigue for two months, along with worsening nausea, vomiting, shortness of breath, and dizziness over the past few days. Initial vitals were within normal limits other than a tachycardic heart rate of 120 beats per minute. No hyperventilation was noted. Physical exam demonstrated dry mucous membranes and scleral icterus. The body mass index at admission was 22.1 kg/m2. On initial intake, basic metabolic panel results suggested an anion gap acidosis with an anion gap of 15 mEq/L and bicarbonate of 13 mmol/L. Initial venous blood gas in the Emergency Department recorded a pH of 7.17. Sequential arterial blood gas showed a primary metabolic acidosis with compensatory respiratory alkalosis. The osmolal gap was calculated to be 28 mOsm/kg. Details of the initial venous and arterial blood gases can be found in Table 1.

**Table 1:** Results of the initial venous blood gas evaluated in the Emergency Department, followed by confirmatory arterial blood gas. Venous blood gas from the day of discharge is also displayed. pCO2: partial pressure of carbon dioxide; HCO3: bicarbonate

Blood Gas Component	Reference Range	Venous Blood Gas Day 1	Arterial Blood Gas Day 1	Venous Blood Gas Day 3
pH	7.38-7.44	7.17	7.265	7.359
pCO2	35-45 mmHg	33 mmHg	21.3 mmHg	45.3 mmHg
HCO3	22-26 mmol/L	12.5 mmol/L	9.7 mmol/L	25 mmol/L

An extensive history was gathered regarding possible toxic ingestions along with screening for metabolic abnormalities. The patient denied ingestion of products containing ethylene glycol or methanol and reported no use of medications or supplements. Screening for salicylates, acetaminophen, and ethanol were all negative. He denied any history of kidney disease. Lactic acid, creatinine, hemoglobin A1c, and glucose were all within normal limits. Notably, serum ketones were too high to be calculated and urine ketones were 80 mg/dL. He had not been taking any SGLT2 (sodium-glucose cotransporter-2) inhibitors. Upon further history, the patient reported recently following a strict ketogenic diet for the last four months. His diet consisted solely of meat and fat, with the avoidance of all carbohydrates. Details of the initial workup can be found in Table 2.

**Table 2:** Results of laboratory testing at the time of admission for evaluation of anion gap metabolic acidosis.

Laboratory Test	Reference Range	Laboratory Value Day 1
Lactic acid	0.5-2.2 mEq/L	1.1 mEq/L
Procalcitonin	<0.05 ng/mL	<0.05 ng/mL
Creatinine	0.70-1.40 mg/dL	1.29 mg/dL
Blood Urea Nitrogen	5-25 mg/dL	11 mg/dL
Ammonia	11-35 mcmol/L	15 mcmol/L
Hemoglobin A1c	4.3-5.6%	5.1%
Glucose	60-100 mg/dL	99 mg/dL
Ethanol	Neg mg/dL	Neg
Salicylates	2.9-9 mg/dL	<10.0 mg/dL
Acetaminophen	10-20 mcg/mL	<10 mcg/mL
Serum Ketones	0.05-0.27 mmol/L	>5 mmol/L
Urine Ketones	neg-trace	>80 mg/dL

Lactated Ringer's/5% dextrose was initiated, and the patient agreed to resume a normal diet while hospitalized. By the morning of hospital day two, his acidosis and symptoms largely resolved, but magnesium and phosphorus were below normal limits. Due to the patient’s presumed previous malnutrition and rapid initiation of a normal diet, these electrolyte abnormalities were attributed to a process resembling refeeding syndrome. His electrolytes normalized with two days of intravenous electrolyte replenishment. Table 3 displays the evolution of his electrolyte abnormalities throughout admission. The patient was discharged on day three after further potassium and phosphorus replenishment and instructed to no longer follow a ketogenic diet.

**Table 3:** Electrolyte values displayed for each day of admission.

Laboratory Test	Reference Range	Laboratory Value Day 1	Laboratory Value Day 2	Laboratory Value Day 3
Potassium	3.5-5.0 mEq/L	3.8 mEq/L	3.7 mEq/L	2.9 mEq/L
Magnesium	1.8-2.4 mEq/L	2.0 mg/dL	1.7 mg/dL	2.0 mg/dL
Phosphorus	2.5-4.5 mg/dL	--	1.4 mg/dL	1.8 mg/dL

## Discussion

This case discusses a patient who presented as euglycemic ketoacidosis in the absence of diabetes or SGLT2 inhibitors. His metabolic acidosis was due to a strict ketogenic diet. Ketogenesis can occur in both physiologic and pathologic processes, including but not limited to states of exercise, starvation, fasting, and diabetes. Ketone bodies are synthesized in the liver as a response to decreased carbohydrate availability, providing an alternative source of energy for the body. These ketone bodies, primarily acetoacetate and beta-hydroxybutyrate dehydrogenase, can then be circulated to the heart and skeletal muscle and converted to adenosine triphosphate (ATP) [2]. Ketogenic diets with severely limited carbohydrate intake can lead to elevated levels of ketone bodies, causing ketoacidosis.

There is growing evidence of both potential harms and benefits in specific patient populations adhering to the ketogenic diet. The populations that may benefit from the ketogenic diet include patients with obesity [3], Alzheimer's disease [4], drug-resistant epilepsy [5], and those undergoing treatment of certain cancers [6]. The recommended ketogenic diet in these settings usually consists of high fats, moderate proteins, and very low carbohydrates approximately divided into 55% to 60% fat, 30% to 35% protein, and 5% to 10% carbohydrates. At the same time, potential harms of the ketogenic diet include impaired bone remodeling in response to exercise [7], along with reduced exercise performance during high-intensity, short-duration activities [8]. Additionally, ketogenic diets with extremely limited carbohydrate consumption, as in this case, can lead to severe metabolic disturbance, similar to starvation ketosis.

Based on previously published case reports, there may be a trend in ketogenic diets triggering severe metabolic acidosis in certain patient populations, specifically those with diabetes [9,10], SGLT2 inhibitor use [11,12], or current lactation [13-15]. However, only one other previously published case vignette reports a similar presentation of hospitalization following a ketogenic diet in a non-lactating, non-diabetic patient. The case describes a patient who presented in metabolic acidosis after three weeks of a strict no-carbohydrate ketogenic diet for weight loss. She recovered by day three in the hospital after receiving 5% dextrose and sliding-scale insulin [16]. This case, along with the presented case, shows that the ketogenic diet has potentially life-threatening risks, even in previously healthy patients with no history of diabetes or current lactation.

## Conclusions

As the popularity of the ketogenic diet and other fad diets grows, physicians should be aware of and prepared to counsel patients on the potential risks associated with these diets. Specifically with the ketogenic diet, emphasis should be placed on continuing to include a small amount of carbohydrates and understanding the evidence-based indications for such a diet. Although certain patient populations such as those with diabetes, SGLT2 inhibitor use, and current lactation may be at increased risk, there are still potential dangers for previously healthy patients.
